# (E)-9-Octadecenoic Acid Ethyl Ester Derived from Lotus Seedpod Ameliorates Inflammatory Responses by Regulating MAPKs and NF-*κ*B Signalling Pathways in LPS-Induced RAW264.7 Macrophages

**DOI:** 10.1155/2022/6731360

**Published:** 2022-01-05

**Authors:** Chuanqi Xie, Shufen Wang, Mingyuan Cao, Wei Xiong, Lei Wu

**Affiliations:** ^1^Institute of Applied Chemistry, Jiangxi Academic of Sciences, Nanchang 330096, China; ^2^Faculty of Food Science and Engineering, Jiangxi Agricultural University, Nanchang 330000, China

## Abstract

Inflammation is generally considered a key risk factor in the progress of several chronic diseases, such as arthritis, gastritis, and hepatitis. Natural products with anti-inflammatory ability have played a great role in the process of overcoming these inflammatory diseases. In this study, we evaluated the anti-inflammatory activities of ten natural compounds derived from lotus seedpod and discovered (E)-9-octadecenoic acid ethyl ester (E9OAEE) inhibited the production of nitric oxide (NO) optimally in lipopolysaccharide (LPS)-induced RAW264.7 macrophages. Furthermore, we explored the effects of E9OAEE on inflammatory responses and the underlying mechanisms in LPS-induced RAW264.7 macrophages. The results indicated that E9OAEE significantly suppressed the production of NO, prostaglandin E2 (PGE2), and tumour necrosis factor-*α* (TNF*α*) in a dose-dependent manner. The protein expression and mRNA levels of inducible nitric oxide synthase (iNOS) and cyclooxygenase-2 (COX2) were inhibited by pretreatment of E9OAEE. Furthermore, E9OAEE restrained the phosphorylation of mitogen-activated protein kinase (MAPKs) family members, ERK, P38, and JNK stimulated by LPS-treated for 30 min and prevented the nuclear translocation of nuclear factor-kappa B (NF-*κ*B) prompted by LPS-treated for 6 h in RAW264.7 macrophages. Taken together, we discovered an anti-inflammatory component from lotus seedpod and identified E9OAEE attenuated the inflammatory response in LPS-induced RAW264.7 macrophages probably by regulating the activation of MAPKs and NF-*κ*B signalling pathways, which would provide some base for the development of new anti-inflammatory drugs.

## 1. Introduction

Inflammation is a series of physiological reactions to exogenous stimuli and conditions, and is considered a key risk factor in the progress of several chronic diseases, including autoimmune diseases, arthritis, obesity, diabetes, and cancer [1, 2]. Recently, researchers have made great efforts to understand the nature of inflammation and developed numerous anti-inflammatory drugs [3–12]. However, the reality that many people in the world are suffering from inflammatory diseases indicates that it is still of great practical significance to further explore new anti-inflammatory drugs. Macrophages are important immune cells closely related to inflammation by regulating the expression of inflammatory proteins and genes such as iNOS and COX2 and the production of proinflammatory cytokines including NO, PGE2, TNF*α*, and interleukin (IL)-6 and IL-1*β*, which can be continuously activated in response to LPS. The uncontrolled inflammatory responses are closely associated with the development of inflammatory diseases. Hence, anti-inflammatory compounds with the ability to inhibit the expression of inflammatory proteins and genes and the production of proinflammatory markers are an efficient way to control inflammatory diseases [13–26].

Lotus (*Nelumbo nucifera Gaertn*) is a perennial aquatic plant, grown in China, India, some African countries, and Japan, and all parts of lotus, such as seeds, flowers, leaves, and stems, can be consumed as foods or medicines. Further studies have shown that there are several bioactive compounds, such as flavonoids, alkaloid, triterpenoid, and polyphenolic acid in lotus plant [27]. (E)-9-octadecenoic acid ethyl ester (E9OAEE) is a natural product isolated from lotus seedpod, which was usually ignored and abandoned in the process of lotus production. Balachandran and his colleagues have reported that E9OAEE was isolated from the marine algae “Sargassum wigitti” and predicted it has the potential against COX2 to exert anti-inflammatory activity [28]. Nevertheless, the actual anti-inflammatory effect of E9OAEE has not been well known.

The LPS-mediated inflammation is known to activate MAPKs and NF-*κ*B signalling pathways. The nuclear transcription factor NF-*κ*B is a prominent regulator of inflammatory responses, which translocates into the cell nucleus and activates the transcription of proinflammatory cytokines after stimulated by LPS. MAPKs is a kinase family, which contains several members, such as ERK, p38, and JNK, which mediates numerous cellular activities, including proinflammatory cytokine production, cell proliferation, differentiation, survival, and apoptosis. As such, these signalling molecules may be the therapeutic targets of anti-inflammatory agents [29].

In this study, the inflammatory model of LPS-induced RAW264.7 macrophages was used to evaluate the anti-inflammatory activity of E9OAEE. The results showed that E9OAEE significantly inhibits the production of NO and PEG2. Furthermore, the protein expressions and mRNA levels of iNOS and COX2 were also reduced by E9OAEE. Additionally, the production of proinflammatory factor TNF*α* was affected by E9OAEE. And E9OAEE regulated the activation of MAPKS signalling pathways, including ERK, P38, and JNK, and the nuclear translocation of nuclear factor-kappa B. Taken together, these results will provide guidance and theoretical basis for the high value-added utilization of lotus seedpod.

## 2. Materials and Methods

### 2.1. The Source of (E)-9-Octadecenoic Acid Ethyl Ester

In this study, (E)-9-octadecenoic acid ethyl ester was isolated from lotus seedpod in our laboratory and the purity of E9OAEE was about 98% determined by high-performance liquid chromatography (HPLC) with a content of 0.158% of dry matter.

### 2.2. Cell Culture

RAW264.7, a mouse macrophage cell line (a gift from Key Laboratory of Pu-er Tea Science, Ministry of Education), was cultured in high glucose DMEM (Biological Industries, Israel) containing 10% fetal bovine serum (FBS) (Natocor, Argentina), and 1% penicillin and streptomycin (Biological Industries, Israel) at 37°C and 5% CO2.

### 2.3. MTT Assay

RAW264.7 cells (5 × 10^4^/well) were seeded onto a 96-cell plate in triplicate for adherence to a final volume of 200 *µ*L. Then, the cells were incubated with or without E9OAEE for 24 h. Then, 20 *µ*L of MTT (5 mg/mL) was added to each well and incubated at 37°C for 4 h. After supernatant removal from the wells, 200 *µ*L of DMSO was added for the dissolution of formazan crystals. The absorbance of each well was read at 492 nm using a microplate reader (Tecan Infinite 200 Pro, Switzerland).

### 2.4. NO Production Assays

RAW264.7 cells (5 × 10^4^/well) were incubated in a 96-cell plate for adherence with a final volume of 200 *µ*L. Then, the cells were initially stimulated with E9OAEE at different concentrations of 6.25, 12.5, 25, and 50 *µ*g/mL for 2 h. Then, LPS (1 *µ*g/mL) (Sigma-Aldrich, USA) was added for 24 h. The production of NO was measured by the accumulation of nitrites in the culture medium, using the colorimetric Griess reaction with slight modifications. The 100 *µ*L of each supernatant medium was mixed with an equal volume of Griess A (0.1%(w/v) N-(1-naphthyl)-ethylenediamine dihydrochloride) and B (1%(w/v) sulphanilamide containing 5%(w/v) H3PO4) (1 : 1) at room temperature for 10 min in the dark. The absorbance was immediately measured at 540 nm.

### 2.5. Enzyme-Linked Immunosorbent Assay

RAW264.7 cells (1 × 10^6^/well) were cultured in a 6-cell plate overnight, and the cells were treated with a range of E9OAEE concentrations (6.25, 12.5, 25, 50 *µ*g/mL) for 2 h and then stimulated with LPS (1 *µ*g/mL) for 24 h. Cell culture supernatants were collected and added into ELISA plates for the determination of PGE2 (R&D Systems, Minneapolis, USA), TNF*α* (BIOSTER, Wuhan, China), IL6 (BIOSTER, Wuhan, China), and IL-1*β* (BIOSTER, Wuhan, China) following the respective manufacturer's instructions. Each sample was tested in triplicate.

### 2.6. Western Blotting

RAW264.7 cells (1 × 10^6^/well) were cultured in a 6-cell plate overnight and then pretreated with E9OAEE (6.25, 12.5, 25, 50 *µ*g/mL) for 2 h and stimulated with LPS (1 *µ*g/mL) for 24 h or indicated time, respectively. Cells were harvested and lysed in radioimmunoprecipitation assay (RIPA) lysis buffer (Solarbio, Beijing, China) on ice for 30 min, and the supernatant was collected. Proteins were quantified using Enhanced BCA Protein Assay Kit (Beyotime, Jiangsu, China), and equal amounts of protein (40 *µ*g) were separated via 10% sodium dodecyl sulfate (SDS)-polyacrylamide gel and then transferred onto PVDF membranes (Millipore, CA, USA). The membranes were blocked with 5% skim milk at room temperature for 1 h and then incubated overnight at 4°C with primary antibodies specific for iNOS (ABclonal, Wuhan, China), GAPDH (Bioworld Technology, Inc, MN, USA), COX2 (Bioworld Technology, Inc, MN, USA), JNK1/2/3 (ABclonal, Wuhan, China), p-JNK (Bioworld Technology, Inc, MN, USA), P38 (ABclonal, Wuhan, China), p-P38 (Bioworld Technology, Inc, MN, USA), ERK1/2 (ABclonal, Wuhan, China), and p-ERK1/2 (ABclonal, Wuhan, China). The membrane was then incubated for an additional 60 min with a goat anti-rabbit lgG/HRP (Bioss, Beiing, China). Then, the membrane was developed using Super ECL Plus kit (US Everbright Inc, Suzhou, China) for imaging with ChemiScope 3000 mini (Clinx, Shanghai, China).

### 2.7. RNA Isolation and Quantitative Real-Time PCR (qRT-PCR) Analysis

Total RNA was isolated from RAW264.7 cells treated with different concentrations of E9OAEE in the presence of LPS (1ug/ml) using TransZol Up reagent (TransGen Biotech, Beijing, China). One microgram of total RNA was subjected to synthesize cDNA using PrimeScript™ First-Strand cDNA Synthesis kit (Takara, Beijing, China). The real-time PCR was performed using qTOWER 3G (Analytic Jena, Jena, Germany) with TB Green® Premix Ex Taq™ II kit (Takara, Beijing, China). The reactions of the samples were then run in triplicate and were normalized to glyceraldehyde 3-phosphate dehydrogenase (GAPDH) as a housekeeping gene. Relative expression differences of PCR results were calculated by the comparative cycle threshold method. PCR was performed with specific primers iNOS, COX2, and GAPDH (Table 1).

### 2.8. NF-*κ*B Activation and Nuclear Translocation Assay

All operations were executed in accordance with the instructions of the NF-*κ*B activation-nuclear translocation assay kit (Beyotime, Jiangsu, China). RAW264.7 cells (1 × 10^6^/well) were cultured in a 6-cell plate overnight, and the cells were pretreated with various concentrations of E9OAEE for 2 h and LPS for an additional 6 h. Cells were washed, then fixed with the stationary liquid for 15 min, and blocked for 1 h at room temperature. The cells were then incubated overnight at 4°C with the primary antibody NF-*κ*B. Following washing, the NF-*κ*B antibody was removed and further incubated with Cy3-conjugated secondary antibody for 1 h and stained with 4′,6-diamidino-2-phenylindole (DAPI) for 5 min. Images were captured using a TS2-FL microscope (Nikon, Tokyo, Japan).

### 2.9. Statistical Analysis

All experiments were performed in triplicate, and the results were expressed as mean ± SEM of the inducted number of experiments. Statistical analysis comparing LPS-stimulated cells to controls was performed using the two-sided unpaired *t*-test. To compare the effect of different concentrations of E9OAEE in LPS-stimulated cells, one-way ANOVA followed by a Dunnett's multiple comparison test was used. The statistical tests were applied using GraphPad Prism, version 8.0.1 (GraphPad Software, San Diego, CA, USA).

## 3. Results

### 3.1. Effects of E9OAEE on the Viability of RAW264.7 Cells

**Figure 1:**
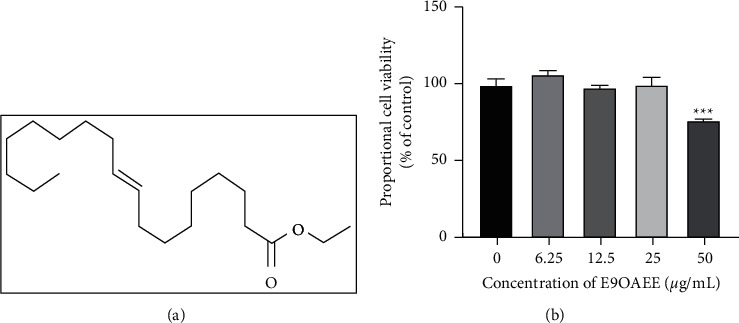
Effects of E9OAEE on the viability of RAW264.7 cells. (a) The chemical construction of E9OAEE. (b) Effects of E9OAEE on the viability of RAW264.7 cells; ^*∗∗∗*^*p* < 0.001 compared with the control group.

In this study, E9OAEE (Figure 1(a)) was first isolated from the lotus (*Nelumbo nucifera Gaertn*) seedpod, which has multiple biological activities. In this study, to investigate the anti-inflammatory potential of E9OAEE, we initially evaluated its effect on the viability of RAW264.7 macrophages using the MTT assay. The results showed that E9OAEE had not any cellular toxicity at 6.25, 12.5, and 25 *μ*g/mL, compared to the control group where E9OAEE was absence, while it inhibited the viability of RAW264.7 cells up to 23% at 50 *μ*g/mL (Figure 1(b)).

### 3.2. Effects of E9OAEE on the Production of Inflammatory Markers in LPS-Induced RAW264.7 Macrophages

**Figure 2:**
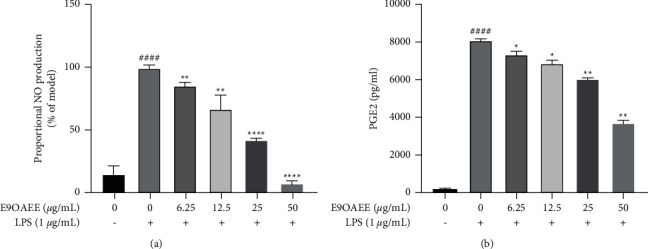
Effects of E9OAEE on the production of inflammatory markers in LPS-induced RAW264.7 cells. (a) Effects of E9OAEE on NO production in LPS-induced RAW264.7 cells. (b) Effects of E9OAEE on PGE2 production in LPS-induced RAW264.7 cells; ^###^*p* < 0.0001 compared with the control group, ^*∗∗∗*^*p* < 0.0001, ^*∗∗*^*p* < 0.01, ^*∗*^*p* < 0.05 compared with the LPS group.

NO and PGE2 are two prominent inflammatory markers in LPS-induced RAW264.7 macrophages. Thus, we next explored the effects of E9OAEE on the production of NO and PGE2. The effect of E9OAEE on NO production was detected using the Griess assay, and the result demonstrated that it significantly reduced the production of NO in a dose-dependent manner (Figure 2(a)). The effect of E9OAEE on the production of PGE2 was examined using ELISA, and the results displayed that it markedly inhibited PGE2 production in a dose-dependent manner (Figure 2(b)). These data suggested that E9OAEE plays a significant role in inhibiting the inflammatory response in LPS-induced RAW264.7 macrophages.

### 3.3. Effects of E9OAEE on the Expression of Inflammatory Proteins in LPS-Induced RAW264.7 Cells

**Figure 3:**
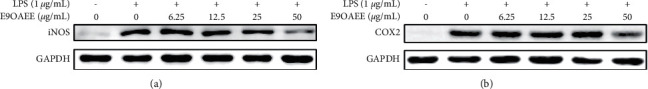
Effects of E9OAEE on the expression of inflammatory proteins in LPS-induced RAW264.7 cells. (a) Effects of E9OAEE on the expression of iNOS protein in LPS-induced RAW264.7 cells. (b) Effects of E9OAEE on the expression of COX2 protein in LPS-induced RAW264.7 cells.

iNOS and COX2 are two key enzymes responsible for the production of NO and PGE2 in LPS-induced RAW264.7 macrophages, respectively. To further evaluate the anti-inflammatory effects of E9OAEE, the protein expression of iNOS and COX2 was analyzed using western blotting. As shown in Figure 3, LPS induced the overexpression of iNOS protein in RAW264.7 macrophages, but E9OAEE attenuated the expression of it in a dose-dependent manner (Figure 3(a)). Similarly, the expression level of COX2 protein also was prompted by LPS and suppressed by E9OAEE in RAW264.7 cells (Figure 3(b)).

### 3.4. Effects of E9OAEE on the mRNA Level of Inflammatory Genes in LPS-Induced RAW264.7 Cells

**Figure 4:**
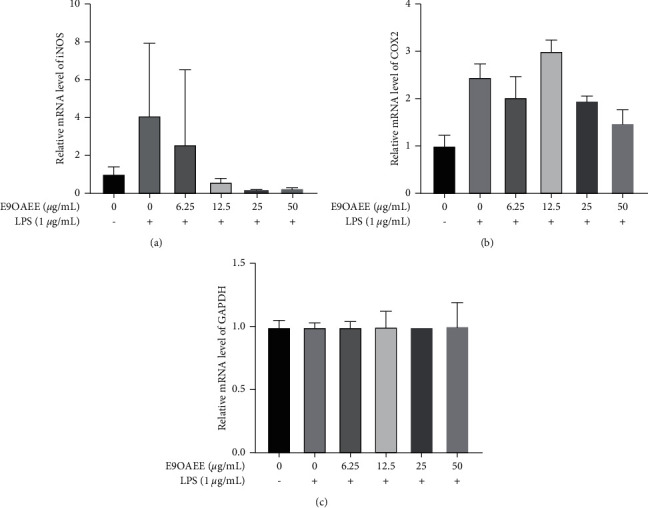
Effects of E9OAEE on the mRNA level of the inflammatory genes in LPS-induced RAW264.7 cells. (a) Effects of E9OAEE on the mRNA level of iNOS in LPS-induced RAW264.7 cells. (b) Effects of E9OAEE on the mRNA level of COX2 in LPS-induced RAW264.7 cells. (c) Effects of E9OAEE on the mRNA level of GAPDH in LPS-induced RAW264.7 cells.

Furthermore, the mRNA expression levels of iNOS and COX2 were examined through RT-PCR. As presented in Figure 4, the mRNA levels of iNOS and COX2 were lower in untreated RAW264.7 cells than in LPS-stimulated cells; however, the pretreatment of E9OAEE reduced the mRNA expression of iNOS and COX2 induced by LPS in RAW264.7 macrophages (Figures 4(a) and 4(b)). The mRNA levels of GAPDH were as internal references (Figure 4(c)).

### 3.5. Effects of E9OAEE on the Production of Proinflammatory Cytokines in LPS-Induced RAW264.7 Macrophages

**Figure 5:**
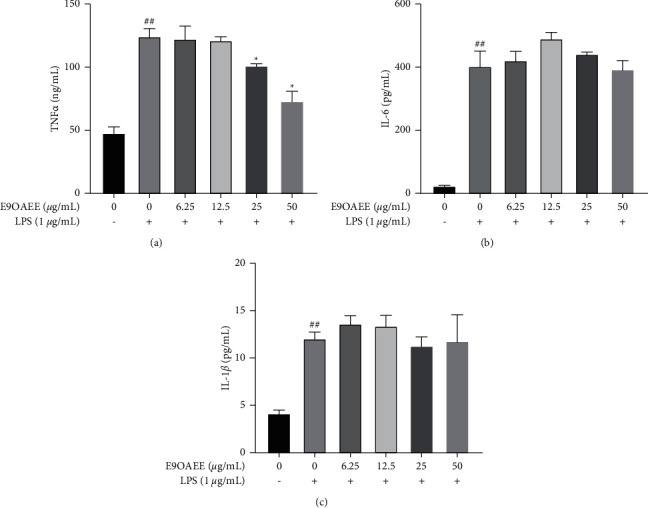
Effects of E9OAEE on the production of proinflammatory mediators in LPS-induced RAW264.7 cells. (a) Effects of E9OAEE on the production of TNF*α* in LPS-induced RAW264.7 cells. (b) Effects of E9OAEE on the production of IL6 in LPS-induced RAW264.7 cells. (c) Effects of E9OAEE on the production of IL-1*β* in LPS-induced RAW264.7 cells; ^##^*p* < 0.01 compared with the control group, ^*∗*^*p* < 0.05 compared with the LPS group.

In addition, proinflammatory cytokines, such as TNF*α*, IL6, and IL-1*β*, are closely associated with the development of inflammation. In this study, we tested the effects of E9OAEE on the production of proinflammatory cytokines in LPS-stimulated RAW264.7 macrophages. As shown in Figure 5, the LPS treatment prominently stimulated the production of TNF*α* in RAW264.7 cells, but the pretreatment of E9OAEE decreased the production of it in a dose-dependent manner (Figure 5(a)). In contrast, the pretreatment of E9OAEE did not reduce the production of IL6 and IL-1*β* induced by LPS in RAW264.7 cells (Figures 5(b) and 5(c)). These data indicated that the anti-inflammatory effects of E9OAEE are intently linked to the production of TNF*α*, but not IL6 and IL-1*β* in RAW264.7 macrophages.

### 3.6. Effects of E9OAEE on the Activation of MAPK Signalling Pathway in LPS-Induced RAW264.7 Macrophages

**Figure 6:**
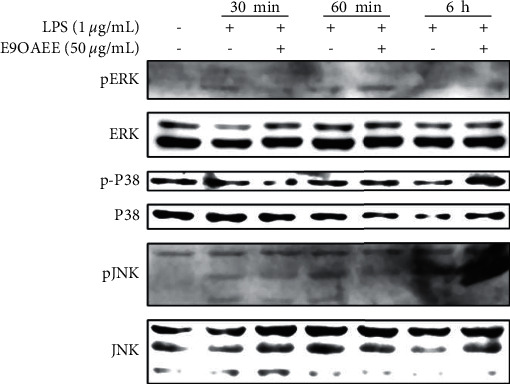
Effect of E9OAEE on the phosphorylation of MAPK signalling pathway in LPS-induced RAW264.7 cells. The phosphorylated proteins and total proteins of ERK, P38, and JNK were presented after treated by E9OAEE and LPS for indicated times.

The LPS-mediated inflammation is known to activate MAPKs signalling pathways. To explore the potential mechanisms underlying the anti-inflammatory activity of E9OAEE in LPS-stimulated RAW264.7 macrophages, the effects of it on the LPS-stimulated phosphorylation of MAPKs were analyzed. As presented in Figure 6, treatment with LPS for 30 min increased the phosphorylation of ERK, p38, and JNK in RAW264.7 cells, but the pretreatment of E9OAEE suppressed the LPS-induced phosphorylation of them. In contrast, E9OAEE did not inhibit the phosphorylation of MAPKs after treated with LPS for 1 h and 6 h. These data suggested that the effects of E9OAEE on the phosphorylation of ERK, p38, and JNK MAPKs were in a short time.

### 3.7. Effects of E9OAEE on the Nuclear Translocation of NF-*κ*B in LPS-Induced RAW264.7 Cells

**Figure 7:**
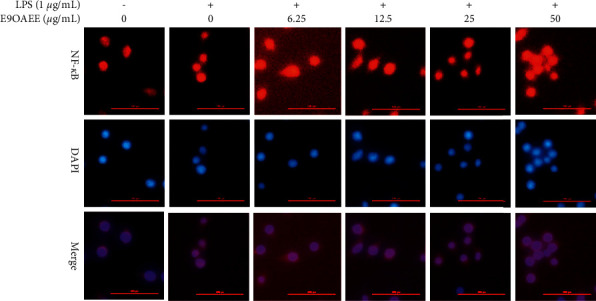
Effects of E9OAEE on the nuclear translocation of NF-*κ*B in LPS-induced RAW264.7 cells. The nuclear translocation of NF-*κ*B in LPS-induced RAW264.7 cells was detected using NF-*κ*B activation nuclear translocation assay kit after treated by E9OAEE and LPS for 6 h.

In addition to activating MAPK signalling pathways, the LPS-mediated inflammation allows NF-*κ*B to translocate into the cell nucleus. And NF-*κ*B is a regulator of the expression of inflammatory proteins, such as iNOS and COX2. To investigate whether E9OAEE was involved in the regulation of NF-*κ*B activation, the nuclear translocation of NF-*κ*B was detected through immunofluorescence assays in LPS-induced RAW264.7 cells. As shown in Figure 7, the fluorescence graph indicated that NF-*κ*B entered into the nucleus from the cytoplasm after treated by LPS for 6 h; however, the process was prevented by the pretreatment of E9OAEE in RAW264.7 macrophages. These data revealed that E9OAEE is a negative regulator of the LPS-induced nuclear translocation of NF-*κ*B in RAW264.7 cells.

## 4. Discussion

In the previous study, Ajay Kumar and his colleagues have predicted that E9OAEE has the potential to be against COX2 to exert anti-inflammatory activity by molecular docking stimulation [28]. However, little is known about the actual inhibitory effects of E9OAEE on inflammatory responses. Here, a canonical inflammatory model of LPS-stimulated RAW264.7 macrophages was recruited to evaluate the anti-inflammatory effects of E9OAEE. The data showed that E9OAEE suppressed the production of inflammatory markers, NO and PGE2, reduced the protein expression and mRNA levels of iNOS and COX2, inhibited the production of proinflammatory cytokine TNF*α*, and regulated the phosphorylation of MAPK family members, ERK, P38, and JNK and the nuclear translocation of NF-*κ*B in LPS-induced RAW264.7 macrophages.

NO and PGE2 as the two critical inflammatory markers are regulated by iNOS and COX2, respectively [13, 17, 20, 29–33]. In the present study, pretreatment with E9OAEE inhibited the LPS-induced production of NO and PGE2 (Figure 2) and proteins and mRNA expression of iNOS and COX2 (Figures 3 and 4) in RAW264.7 cells. These findings suggest that E9OAEE has anti-inflammatory effects against LPS-induced inflammatory responses. In addition, RAW264.7 macrophages stimulated by LPS produce several proinflammatory cytokines, including TNF*α*, IL6, and IL-1*β* [13]. In this investigation, we examined whether E9OAEE inhibits the LPS-induced production of TNF*α*, IL6, and IL-1*β*, and the results demonstrated that pretreatment with E9OAEE significantly inhibited the LPS-induced TNF*α* production in RAW264.7 macrophages, but not IL6 and IL-1*β* (Figure 5). This indicates that the anti-inflammatory activity of E9OAEE may be associated with the inhibition of TNF*α* production in RAW264.7 macrophages.

Mechanically, the LPS-mediated inflammation is known to activate MAPKs and NF-*κ*B signalling pathways [29]. MAPKs are important signalling pathways associated with the regulation of cell growth, proliferation, differentiation, migration, inflammation, and survival [13]. The three members of MAPKs consist of the extracellular signal-regulated kinases (ERKs), c-Jun N-terminal kinase (JNK), and p38. Many investigations have demonstrated that candidate materials derived from natural products exhibit anti-inflammatory effects by regulating the activation of MAPK signalling pathways. Therefore, it is possible that the anti-inflammatory mechanisms of E9OAEE are involved in the inhibition of MAPK signalling pathway in LPS-induced RAW264.7 macrophages. Expectedly, our results revealed that E9OAEE inhibited the phosphorylation of MAPK signalling pathways, including ERK, p38, and JNK in the 30-min treatment, while not in longer times (Figure 6). This maybe suggests that the effects of E9OAEE on the phosphorylation of MAPKs are instantaneous. In addition, NF-*κ*B is a vital transcription factor regulating several aspects of inflammatory responses, including gene and protein expression [13]. The inactivation of NF-*κ*B signalling pathway is a significant strategy for the treatment of inflammatory disorders. In this study, the pretreatment of E9OAEE inhibited the nuclear translocation of NF-*κ*B, which was prompted by LPS treatment for 6 h (Figure 7). This indicates that the suppression effects of E9OAEE on the inflammatory responses in LPS-induced RAW264.7 macrophages are probably related to the inhibition of NF-*κ*B nuclear translocation.

Taken together, this study identified the anti-inflammatory effects of E9OAEE on LPS-stimulated RAW264.7 macrophages, which would provide a basis for the development of new anti-inflammatory drugs.

## 5. Conclusion

In this study, our data indicate that E9OAEE exhibits anti-inflammatory effects by reducing the production of NO, PGE2, and TNF*α* and suppressing the expression of protein and mRNA levels of iNOS and COX2 in LPS-induced RAW264.7 macrophages. These inhibitory effects of E9OAEE might be associated with the inactivation of MAPKs (ERK, P38, and JNK) and NF-*κ*B signalling pathways.

## Figures and Tables

**Table 1:** PCR primers used in this study.

Primer name	Primer sequence
iNOS-F	CCCTTCCGAAGTTTCTGGCAGCAGC
iNOS-R	GGCTGTCAGAGCCTCGTGGCTTTG
COX2-F	AGAAGGAAATGGCTGCAGAA
COX2-R	GCTCGGCTTCCAGTATTGAG
GAPDH-F	CACTCACGGCAAATTCAACGGCA
GAPDH-R	GACTCCACGACATACTCAGCAC

## Data Availability

The data used to support the findings of this study are available from the corresponding author upon request.
